# Functional activity level reported by an informant is an early predictor of Alzheimer’s disease

**DOI:** 10.1186/s12877-023-03849-7

**Published:** 2023-03-31

**Authors:** Alexandra Vik, Marek Kociński, Ingrid Rye, Astri J. Lundervold, Alexander S. Lundervold

**Affiliations:** 1 Mohn Medical Imaging and Visualization Centre (MMIV), Department of Radiology, Haukeland University Hospital, Bergen, Norway; 2 Department of Biomedicine, University of Bergen, Bergen, Norway; 3 Department of Biological and Medical Psychology, University of Bergen, Bergen, Norway; 4 Department of Computer Science, Electrical Engineering and Mathematical Sciences, Western Norway University of Applied Sciences, Bergen, Norway

**Keywords:** Alzheimer’s disease, Classification, Explainable machine learning, Functional activity questionnaire, Mild cognitive impairment, Partial dependency plots, SHapley Additive exPlanations

## Abstract

**Background:**

Loss of autonomy in day-to-day functioning is one of the feared outcomes of Alzheimer’s disease (AD), and relatives may have been worried by subtle behavioral changes in ordinary life situations long before these changes are given medical attention. In the present study, we ask if such subtle changes should be given weight as an early predictor of a future AD diagnosis.

**Methods:**

Longitudinal data from the Alzheimer’s Disease Neuroimaging Initiative (ADNI) were used to define a group of adults with a mild cognitive impairment (MCI) diagnosis remaining stable across several visits (sMCI, *n*=360; 55-91 years at baseline), and a group of adults who over time converted from having an MCI diagnosis to an AD diagnosis (cAD, *n*=320; 55-88 years at baseline). Eleven features were used as input in a Random Forest (RF) binary classifier (sMCI vs. cAD) model. This model was tested on an unseen holdout part of the dataset, and further explored by three different permutation-driven importance estimates and a comprehensive post hoc machine learning exploration.

**Results:**

The results consistently showed that measures of daily life functioning, verbal memory function, and a volume measure of hippocampus were the most important predictors of conversion from an MCI to an AD diagnosis. Results from the RF classification model showed a prediction accuracy of around 70% in the test set. Importantly, the post hoc analyses showed that even subtle changes in everyday functioning noticed by a close informant put MCI patients at increased risk for being on a path toward the major cognitive impairment of an AD diagnosis.

**Conclusion:**

The results showed that even subtle changes in everyday functioning should be noticed when reported by relatives in a clinical evaluation of patients with MCI. Information of these changes should also be included in future longitudinal studies to investigate different pathways from normal cognitive aging to the cognitive decline characterizing different stages of AD and other neurodegenerative disorders.

**Supplementary Information:**

The online version contains supplementary material available at 10.1186/s12877-023-03849-7.

## Introduction

Loss of autonomy in everyday life activities is a core symptom of neurodegenerative diseases, including Alzheimer’s disease (AD). It affects a wide range of activities of daily living (ADL), such as managing finances and medication, running errands, preparing meals, and maintaining interests and abilities to take part in already established hobbies [1]. In patients with AD, this deprivation of autonomy is closely related to a cognitive impairment that gradually tends to worsen [2].

Although the diagnostic criteria for the dementia syndrome of AD are not met until a patient shows a major functional disability in these ADL activities, more subtle alterations in ADL functions may raise concerns and worries both by patients and their relatives at a much earlier stage. The concept of mild cognitive impairment (MCI) is used to describe a stage between normal cognitive aging and the more severe decline of dementia characterizing patients with AD [3]. The importance of changes in ADL functions even at this stage is supported by studies showing their impact on the rate of subsequent progression to a more severe dementia syndrome [4–7]. These results point to the value of assessing everyday functioning as part of general practitioner’s (GP’s) consultations when a patient presents symptoms suspected of being early signs of a trajectory towards an AD diagnosis [8, 9].

In fact, both ADL alternations and cognitive symptoms tend to manifest themselves several years after the pathology is well established in the brain [10]. One of our previous studies [11] included performance on a set of cognitive tests and brain measures derived from a magnetic resonance imaging (MRI) examination as features to predict future AD in patients with an MCI diagnosis. When analyzed within a machine learning (ML) framework, the results showed the value of including both features as predictors. In the present study, we follow up on these results by adding information about daily life functioning as a feature in our prediction model, and by running post hoc analyses to build trust in the results generated from the prediction model and to improve the clinical interpretability of the results. By this, we aimed to investigate the value of even subtle changes in ADL activities as a predictor of a trajectory towards the more global dementia syndrome characterizing patients with AD.

Longitudinal data from the Alzheimer’s Disease Neuroimaging Initiative (ADNI) were used to define two groups: one group including adults with an MCI diagnosis that remained stable across all registered visits (sMCI), and a group of adults that over time converted from an MCI to an AD diagnosis (cAD). Daily life functioning was defined from reports on the functional activity questionnaire (FAQ). These reports were included in a Random Forest (RF) classification model together with performance on a set of psychometric tests of memory and executive function and MRI-derived brain measures of the volume of hippocampus and the lateral ventricle volumes (LVV). In addition to presenting model performance, comprehensive model interpretation was applied, including post hoc algorithms such as Shapley Additive exPlanation (SHAP) values and partial dependency plots (PDP). From previous studies, we expected that changes in daily life activities would consistently be among the features with the strongest importance [7] and that this importance would be shared by performance on tests of memory function and related brain structures (e.g., [11, 12]). Ultimately, by including two carefully selected groups of patients with MCI and analytic models, we aimed to contribute to the task of obtaining personalized prognostic information for patients at risk of developing a neurodegenerative disease.

## Methods

### Data set

#### The ADNI cohort

Data used in the preparation of this study were obtained from the Alzheimer’s Disease Neuroimaging Initiative (ADNI) database (adni.loni.usc.edu). The ADNI was launched in 2003 as a public-private partnership, led by Principal Investigator Michael W. Weiner, MD. The primary goal of ADNI has been to test whether serial magnetic resonance imaging (MRI), positron emission tomography (PET), other biological markers, and clinical and neuropsychological assessment can be combined to measure the progression of mild cognitive impairment (MCI) and early Alzheimer’s disease (AD). The study was approved by the institutional review boards of all participating ADNI centers. Written informed consent was obtained from all participants or authorized representatives after an extensive description of the ADNI study according to the Declaration of Helsinki. All methods were performed in accordance with relevant guidelines and regulations [13].

#### ADNI diagnose criteria

At inclusion, an MCI diagnosis was defined according to the following criteria: the presence of (1) cognitive complaints, either reported by the participant, an informant, or a clinician; (2) objective memory impairment defined as a score below the education-adjusted cut-off on the Wechsler Memory Scale Revised, Logical Memory II sub-scale (delayed recall); (3) a Mini-Mental State Examination (MMSE) score between 24 and 30; (4) a clinical Dementia Rating (CDR) of 0.5; and (5) preserved functional abilities such that criteria for dementia diagnosis were not fulfilled. AD diagnosis was based on the NINCDS/ADRDA criteria for probable AD, including memory complaint verified by a study partner, impaired memory function (defined as for MCI); MMSE scores between 20-24 and a CDR scale score of 0.5 or 1.0. All diagnoses were made without the use of MRI scans or other biomarkers. Further details on the inclusion and exclusion criteria used in the ADNI are given in the clinical protocols (http://adni.loni.usc.edu/methods/documents/).

#### Subsample

The present study included results from a baseline assessment when all participants were diagnosed as MCI. Longitudinal data were used to define the following two groups (based on the ADNI-1, ADNI-2, ADNI-GO and ADNI-3 phases): a group of patients where the MCI diagnosis remained stable across all visits (mean 4.5 years follow-up) (sMCI) and a group of adults converting from MCI to AD at some time point during follow-up (cAD). All patients in either group were defined as MCI and AD, respectively, at the final time-point. A total of 708 patients were selected for the current study. After removing missing values, we were left with 360 patients defined as sMCI and 320 defined as cAD. Demographic information for the two subgroups is displayed in Table 1. The data for the present study was downloaded in September 2020.

**Table 1:** Demographics, cognitive and functional characteristics on the included subsample extracted from the ADNI cohort. Total number of subjects, gender distribution, mean, range and standard deviations (SD) of age, education level, length of participation and functional and cognitive measures are given separately for the training and test sets in the two subgroups (sMCI and cAD)

	sMCI (360)	cAD (320)
	Train (285)/Test (75)	Train (255)/Test (65)
**Demographics**		
Sex (F:M)	114:171/32:43	99:156/25:40
Age at inclusion [years]: mean (SD)	73.9 (7.4)/72.7(7.3)	73.9 (7.7)/73.9 (6.9)
Age at inclusion [years]: range	55-91/57.8-87.8	55.2-88.3/55-88.4
Education [years]: mean (SD)	15.8 (2.9)/16.2(2.9)	15.8 (2.9)/16.2(2.9)
Participation length [years]: mean (SD)	4.6 (2.8)/4.5(2.7)	5.0 (2.7)/5.5(2.8)
**Cognitive function**		
RAVLT immediate recall: mean number (SD)	36.9(10)/36.8(9)	28.8(7.8)/30.7(6.9
RAVLT delayed: mean number (SD)	4.9(3.9)/4.6(3.6)	1.9(2.6)/2.4(2.7)
RAVLT recognition: mean number (SD)	11.2(3.2)/11.4(3.0)	9.3(3.5)/9.8(3.6)
TMTA: mean seconds (SD)	39(16)/38(12)	44(20)/44(26)
TMTB: mean seconds (SD)	109(60)/102(44)	134(72)/132(81)
CFT animals: mean number (SD)	17.7(5.1)/18.1(5.0)	15.8(4.9)/15.6(4.3)
**Functional level**		
FAQ Total: mean (SD)	1.9(2.9)/2.4(4.6)	4.9(4.5)/4.5(4.6)
GDS: mean (SD)	1.6(1.5)/1.8(1.3)	1.7(1.4)/1.4(1.2)

### Cognitive, functional and MRI measures

#### Cognitive measures

Results on six psychometric tests were available at baseline. Three scores assessing different aspects of verbal memory function were derived from the Rey Auditory Verbal Learning Test (RAVLT): immediate recall (RAVLT-Im, number of correct words recalled across the immediate recall of the five learning trials); delayed recall (RAVLT-Delay, number of correct words recalled at a 30-minute delayed free recall trial); and recognition (RAVLT-Recognition, number of words correctly recognized in a recognition trial). Three measures assessing aspects of executive function were obtained from the time to complete parts A and B of the Trail Making Test (TMT) and the number of correct unique names reported on the Category Fluency Test (CFT, animals). These variables were selected because they were administered to a large fraction of ADNI participants. In addition, the total score from the geriatric depression scale (GDS) was acquired from the initial screening procedure of ADNI (GDS file) [14].

#### Reports on the functional activity questionnaire (FAQ)

The ability to independently perform activities of daily life (ADLs) was evaluated by asking relatives to complete the FAQ [15]. The questionnaire includes 10 items assessing functions related to the following everyday tasks: (1) writing checks, balancing a checkbook, and paying bills **(FAQ Bills)**; (2) organizing tax, business, and insurance papers **(FAQ Taxes)**; (3) shopping for clothes, household goods, and groceries **(FAQ Shopping)**; (4) playing a game of skill such as chess or bridge or working on a hobby **(FAQ Games)**; (5) heating water for coffee/tea and turning off the stove **(FAQ Beverage/stove)**; (6) preparing a meal **(FAQ Meal)**; (7) following current events **(FAQ Events)**; (8) understanding a TV show, book, or magazine **(FAQ Pay attention)**; (9) remembering appointments, family events, and medications **(FAQ Remember Dates)**; and (10) planning travel away from the home by car or bus **(FAQ Travel)**. Each item is rated as follows: 0 (can complete independently/normal), 1 (has difficulty but performs task independently), 2 (requires assistance), or 3 (completely dependent upon a caregiver). The informant could also add the following information on each item: “Never did but could do now” coded as 0 or “Never did and would have difficulty now” equals 1. A sum score across all items ranges from 0 to 30, with higher scores reflecting less independence in ADL activities. A cut-off score of 9 (evaluated to be dependent in three or more activities) is recommended to indicate impaired function [15]. If the subject had never done one of these activities, the answer was coded as “not applicable” and excluded from the analyses conducted in the present study.

#### MRI measures

A standardized and well-described acquisition protocol developed by ADNI was to be followed at each of the multiple ADNI sites. MRI acquisition was applied on MR scanners of field strength 1.5 Tesla in ADNI-1 and 3.0 Tesla in the study phases ADNI-GO, ADNI-2, and ADNI-3. See http://adni.loni.usc.edu/methods/mri-analysis/mri-acquisition for details. The ADNI consortium made available the results from processing the MRI data using FreeSurfer, but they used two different versions of the software (v.4.3 and v.4.1). It is well known that using different versions of FreeSurfer may lead to discrepancies in atrophy estimates [11]. We therefore re-processed all included T1-Weighted MRI images by using the longitudinal stream of FreeSurfer v.7.1 [16] before entering the analyses of the present study. In the longitudinal stream, an unbiased within-subject template space and image [17] is created using robust, inverse consistent registration [16]. Several processing steps are included to improve reliability and statistical power [16], such as skull stripping, Talairach transforms, atlas registration, as well as spherical surface maps and parcellations that are then initialized with common information from the within-subject template. To reduce the effect of individual and gender differences in brain size, we normalized the volumes using the total intracranial volume measure estimated (eTIV) by FreeSurfer. Two brain measures were derived from the MRI examinations: volume measures of the hippocampus, regarded as a hallmark region for memory loss in neurodegenerative disease [18, 19], and the lateral ventricle volumes (LVV), used as a proxy of brain tissue volume [20–22]. We combined the volumes from the left and right hemispheres to improve the robustness of the measure.

### Prediction of sMCI versus cAD

#### Training and test datasets

**Fig. 1:**
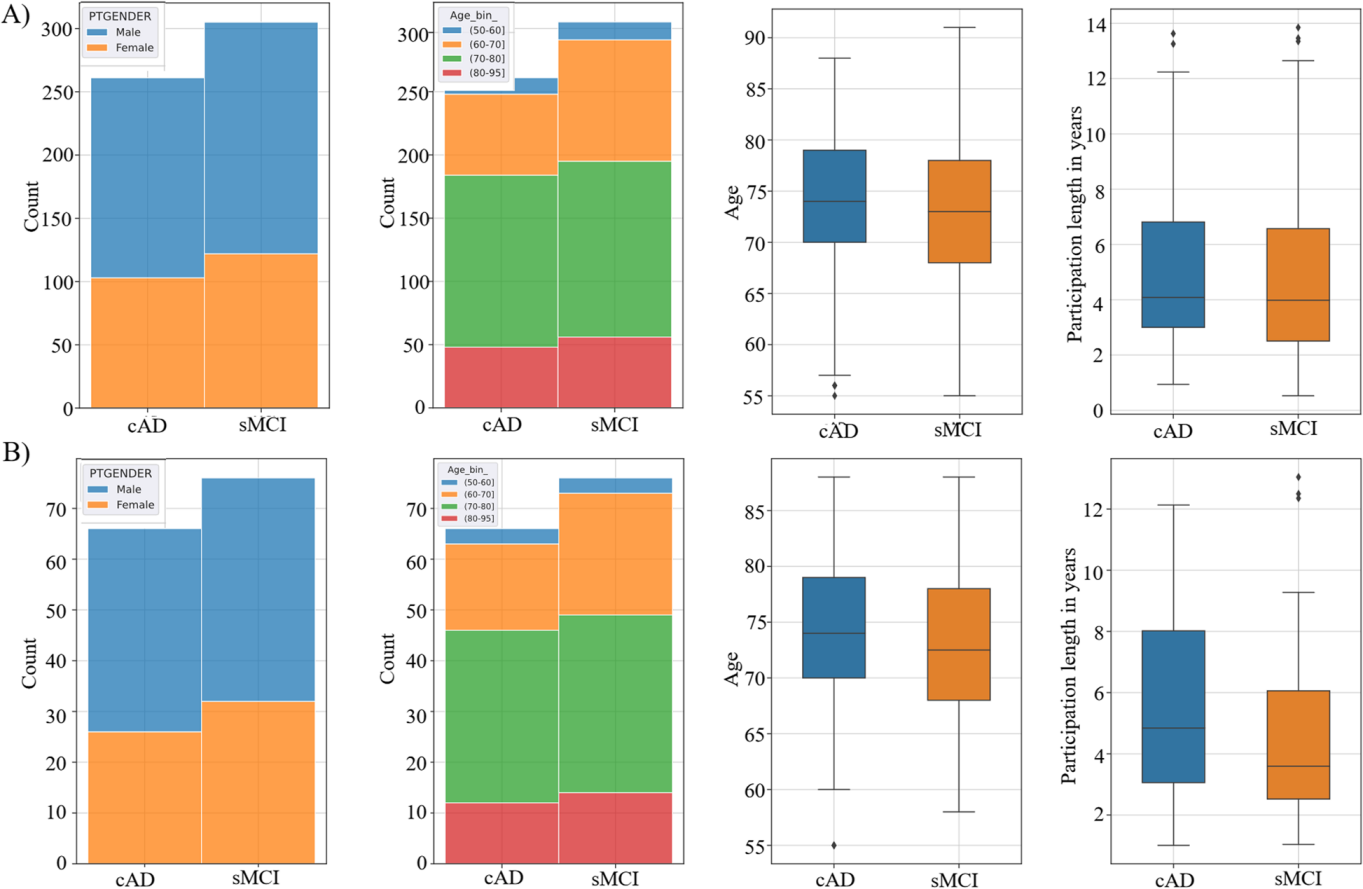
Train-Test Balance. Controlling for gender, age bins, age and length of participation in years in the train (**A**) and test (**B**) set

A machine learning framework was used to classify subjects as belonging to one of the two pre-defined groups (sMCI and cAD). To assess the generalization ability of our predictive models, we put aside a test set containing 21% of the subjects (*n*=140). Thus 79% of the participants formed the training dataset (*n*=540). The train and test sets were balanced by stratifying the two test sets with regard to age-bins, gender, and class belonging (Fig. 1). See Table 1 for information of demographics, cognitive test performances and functional ADL level of the subjects in the two datasets. None of the subjects were present in both the train- and test sets.

#### Random forest

For predicting those defined as sMCI versus those converting to AD, we applied a random forest algorithm [23] as implemented in scikit-learn. A random forest is a meta-estimator that fits several decision tree classifiers on various sub-samples of the data set and uses averaging to improve predictive accuracy and combat overfitting. To improve model performance, we conducted a grid search using 10-fold cross-validation to identify model hyperparameters. The best parameters revealed by the search were utilized in the classifier: *n_estimators=65*, *criterion=‘gini’*, and *max_features=‘2’*; *min_samples_leaf=2*; *min_samples_split=2*; *max_dept=6*.

To assess the model performance, we computed an accuracy (ACC), precision (PRE), recall (REC), and F1-score (F1), calculated for each fold and each classifier. To further assess the model performance, a 2x2 confusion matrix was constructed, displaying the true labels versus the classification labels returned from the prediction on the hold-out validation set. To estimate the relative importance of the multimodal features, we calculated their importance score (range 0-1) using the mean decrease in impurity (Gini), as implemented in scikit-learn.

#### Model interpretation

To clinically implement prediction models, they must be accurate as well as interpretable [24]. The present study therefore applied a diverse range of algorithms to enable a comprehensive evaluation of the models’ decision behavior. Permutation importance, also called mean decrease accuracy, was applied with an in-house algorithm that can group potentially correlated features, inspired by the rfpimp R-package available via https://github.com/parrt/random-forest-importances. Permutation importance is a robust data-driven technique assessing the relevance of features based on measuring the decrease in model accuracy when each feature is randomly shuffled multiple times. In the present study, permutation was tested with 2000 repetitions for each feature or group of features. The negative impact on performance when permuting an important feature is larger than for less important features [23]. Additionally, we implemented a drop-feature setup based on the idea that a feature is important for a model if dropping the feature reduces the model’s performance. The importance score is defined as the re-trained model performance reduction, applied iteratively for one of the feature columns at a time (either single or grouped features). The permutations were shuffled 2000 times, and we reported the average importance values.

The specific effect on the prediction model within the range of values of each feature was investigated via partial dependency plots (PDP) and individual composition expectation (ICE) plots. Partial dependence plots display the response for a single feature in the model while holding all other features constant [25]. Importantly, the plots can capture whether relationships between a feature or a set of features and specified targets are linear or more complex [25]. While PDP plots illustrate general effects across the cohort, the ICE plots display the prediction changes for each individual. Consult [24, 25] for further details.

The SHapley Additive exPlanations (SHAP) technique proposed by Lundberg and Lee [24, 26] was included as a third permutation-based method to investigate feature importances. While permutation feature importance is based on the decrease in model performance, SHAP values are based on the magnitude of feature attributions. Thus, a Shapley value for a given feature value can, in a prediction model framework, be interpreted as the difference between the actual prediction and the average prediction for the whole dataset. We presented SHAP values visualized in a summary plot as it combines feature importance with feature effects. Hence, the plots will both show the direction and degree of influence on the model decision through the SHAP value of each variable and the sum of the absolute SHAP values. Furthermore, we also included a SHAP auto-cohort feature explanation, utilizing a DecisionTreeRegressor from scikit-learn and analyzed interaction effects between the included features.

#### Implementation

We used Python (v. 3.7.6), Jupyter Notebook, and the Python ecosystem for data science for our implementations. Figures 1, 3, 4, 5, 7B were produced using Matplotlib, while Figs. 6 and 7A were produced using pdbox.

## Results

### Predicting sMCI versus cAD - random forest model performance

Reports on FAQ, performance on cognitive tests, and brain measures derived from MRI examinations were included as features in an RF classification model with a k-fold (k=10) grid search cross-validation procedure. When the trained model was tested on unseen data, predictions of class belongings were above chance level. In this hold-out test set the RF classifier obtained an accuracy, recall, precision, and F1 score of 73%, 69%, /72% and 70%, respectively.

**Fig. 2:**
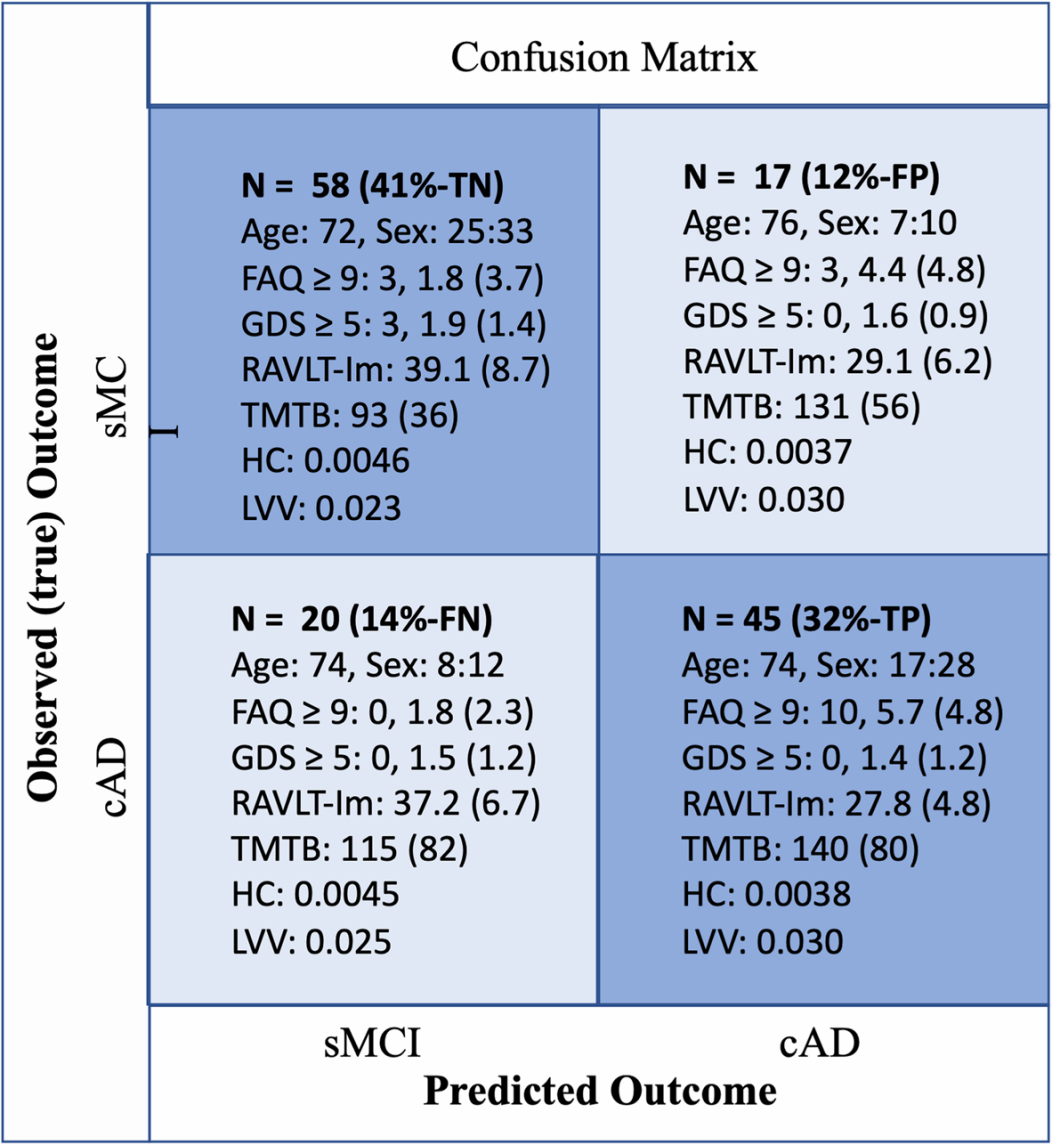
Confusion Matrix. The 2 $\times$ 2 confusion matrix computed for the sMCI and cAD labels returned from applying the trained nonlinear RF model prediction on the test set compared with the co-occurrences of the true (observed) sMCI/cAD (longitudinal defined diagnose) labels. The diagonal cells represent correctly classified subjects (the number of occurrences in each cell is given as N, TN: true negative, TP: true positive, FP: false positive, FN: false negative), and these cells are shaded in blue. Off-diagonal cells represent various events of misclassification. Observed/predicted co-occurrences are also accompanied, for each cell, with corresponding information about sex ratio (F/M), mean(SD) in; FAQ: Functional Activity Questioner, GDS: Geriatric Depression Scale, RAVLT-Im: Rey Auditory Verbal Learning Test immediate recall, TMTB: Trail Making Test part B, HC: hippocampus volume, LVV: lateral ventricle volume

We obtained single-subject predictions from the unseen test set, enabling detailed characteristics of the subgroups of correctly and wrongly classified patients. This information is described within each cell of the confusion matrix presented in Fig. 2 (see Table S1 for details). The model misclassified $\frac{17}{75}$ sMCI subjects as cAD and $\frac{20}{65}$ cAD subjects as sMCI, resulting in a sensitivity of 69% and specificity of 77%. Compared to the wrongly classified sMCI subgroup (14% - false negative), the correctly classified sMCI subgroup (41% - true negative) had on average lower age, higher performance on cognitive tests, a larger volume of the hippocampus, and lower volumes of the lateral ventricles.

Furthermore, the correctly predicted cAD subgroup (32% - true positive) were characterized by lower performance on cognitive tests and larger hippocampal volume compared to the cAD subgroup that was falsely predicted as sMCI by the model (14% - false negative.) In addition, subjects within the correctly predicted cAD subgroup were also reported with the largest decline in everyday life functioning, and a large proportion within this subgroup had a FAQ score equal to or above 9. None of the 20 subjects with cAD misclassified as sMCI were rated above this cut-off. Some findings on the depression scale should also be noted. Only three participants obtained a score of 5 on the GDS, and they were all allocated to the correctly classified sMCI subgroup.

**Fig. 3:**
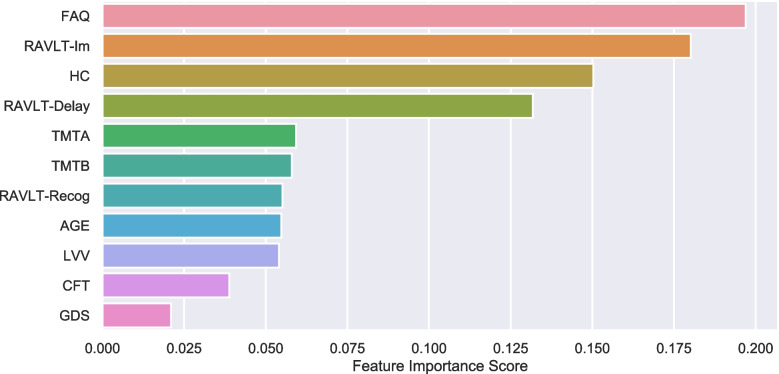
Feature Importance. Bar graph displaying the relative order of the eleven features (y-axis) when classifying sMCI versus cAD by the RF model evaluated on the hold-out validation set. The importance is estimated as gini importance. The x-axis shows the relative importance score. RAVLT: Rey Auditory Verbal Learning Test, TMT: Trail Making Test part A and B, CFT: Category Fluency Test; LVV: lateral ventricle volumes, GDS: Geriatric Depression Scale, FAQ: Functional Activity Questioner

The ranking of the features in the RF model was estimated using *gini importance*. Figure 3 displays the results from the ranking in the hold-out test dataset. The relative order of the eleven features shows that the FAQ was ranked as the most important feature. The importance of FAQ was closely followed by high values for immediate memory recall, hippocampus volume, and delayed memory recall.

### Feature contribution: post hoc interpretation methods

We included a comprehensive analytic approach to evaluate the contributions of features generated from the RF prediction model.

**Fig. 4:**
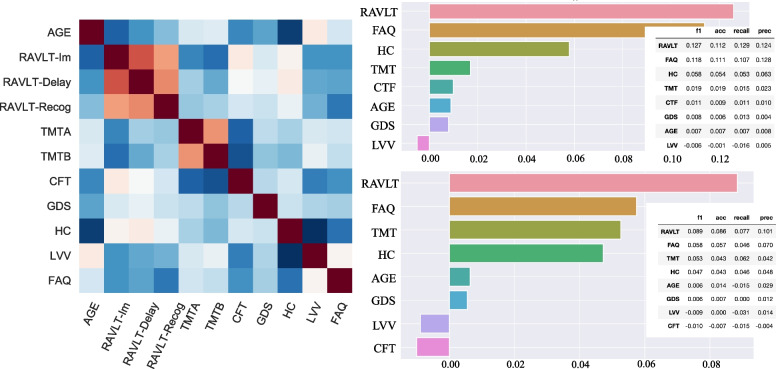
Permutation importance with grouping of correlated features. Displays permutation importance (right upper panel) and the drop-feature importance (right lower panel). Results are reported by taking the average across 2000 repetitions. The importance score (x-axis) is illustrated by the F1-score in the graphs, whereas the complete model evaluation table (accuracy, recall, precision, and F1 scores) is superimposed. Due to multicollinearity, illustrated in the correlation matrix (left panel), RAVLT subscores (Im, Delay and Recog) were grouped: RAVLT, and also for the two parts, A and B, of the TMT in the presented permutation results (right upper and lower panels)

Figure 4 displays permutation importance (right-upper-panel) and drop-feature importance (right-lower-panel) for feature estimations. Correlated features were grouped to avoid poor performance due to the presence of multicollinearity (see the pairwise Pearson’s correlation matrix shown as a heat map in the left panel of Fig. 4). Thus, RAVLT subscores (immediate, delay, and recognition) and the two parts of TMT (A and B) were grouped and named RAVLT and TMT in the permutation importance and drop-feature importance analyses. The permutation importance algorithm measures how much the importance score decreases when a variable is shuffled, breaking any prior relationship between the variable and the target. RAVLT, FAQ, and hippocampus volume were identified with the highest permutation importance, and thereby with a ranking similar to the one generated by the RF model (Fig. 3). The remaining features showed less predictive power, and the LVV showed negative values, indicating that the ventricle volumes did not provide important additional information in this predictive model. Furthermore, the drop-feature importance also emphasized the importance of RAVLT and FAQ for the model prediction. In this model, however, TMT was given a prediction power at the same level as hippocampus volume. The drop-feature importance analysis still left LVV with negative values.

**Fig. 5:**
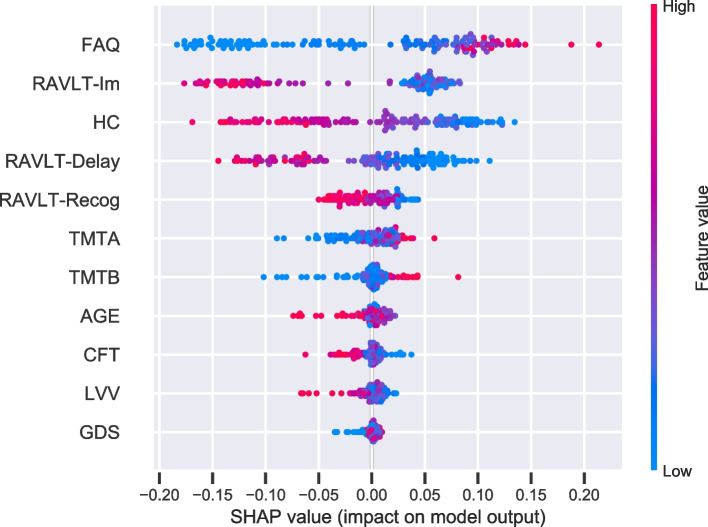
SHAP importance. The figure displays the SHAP summary plot of the features of the RF model. A dot is created for each feature attribution value for the model of each subject. Dots are colored according to feature values. Thus, higher values (represented by red color) for FAQ and lower values for RAVLT -Im, -Delay, and HC (blue) increase the prediction of conversion to AD. Symmetrically, low values in FAQ and high values in RAVLT and HC decrease the prediction of conversion to AD. When the distribution is clustered around 0 indicates that the feature is less relevant. The more skewed the distribution, the more important the feature. The features are ordered according to their importance

While permutation feature importance is based on the decrease in model performance, SHAP values were included to investigate the magnitude of feature attributions. Figure 5 illustrates feature importance based on estimates of SHAP values. Also, here, the FAQ was given the strongest importance. The summary plot combines feature importance with feature effects (Fig. 5). Each point on the summary plot is a SHAP value for a feature and an instance. The position on the y-axis is determined by the feature and on the x-axis by the SHAP value. Thus, the results show that higher values (represented by red color) for FAQ and lower values for immediate- and delayed -recall and hippocampus volume (blue) increase the strength of the prediction to convert to AD. Symmetrically, low values in FAQ and high values in RAVLT and hippocampus decrease the prediction of conversion from a mild to the more severe impairment characterizing the dementia of AD.

Taken together, the three feature importance results, permutation, drop-feature, and the SHAP summary-plot, support the relevance of the features defined by the default RF model, suggesting that measures of everyday functioning, verbal memory, and hippocampus volume should be given weight in a model predicting conversion from MCI to an AD diagnosis.

**Fig. 6:**
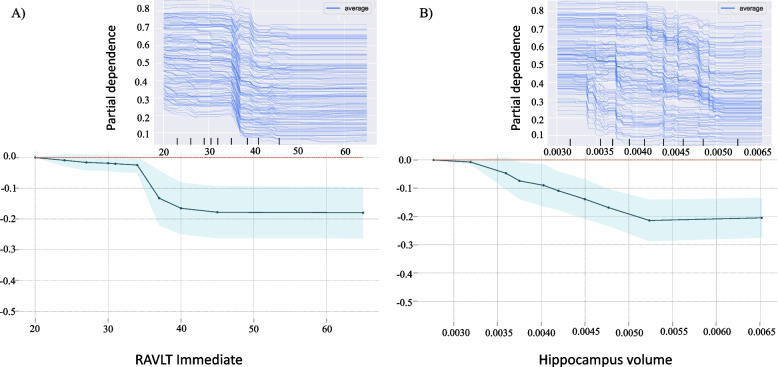
The dependence of the prediction on a single features. Illustrates the marginal effect of RAVLT Immediate (**A**) and hippocampus volume (**B**), have on our RF model. The X-axis represents the range of the feature, and the Y-axis shows changes in the prediction. Positive values represent the contribution of the feature to the increase in the odds to convert to AD. The shaded area represents the standard deviation. The same effects can be observed in the ICE plot and the PDP for RAVLT recall (**A**). However, the ICE plot for the hippocampus shows a skewed tendency (**B**)

**Fig. 7:**
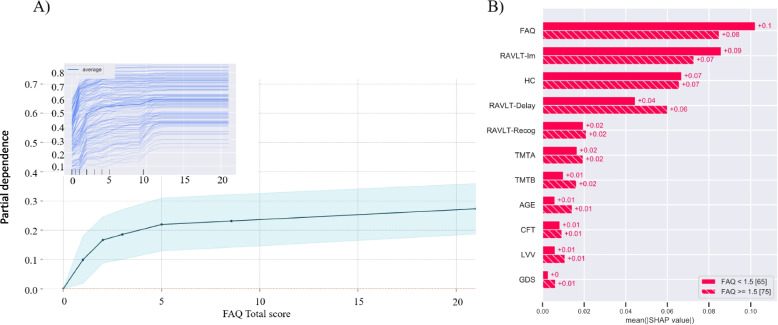
Model exploration: PDP of the FAQ and SHAP auto-cohort split. **A** Illustrates the marginal effect the FAQ total score have on the RF model. The X-axis represents the range of FAQ values (0-30), and the Y-axis shows changes in the prediction. Positive values represent the contribution of the FAQ to the increase in the odds to convert to AD. The shaded area represents the standard deviation. The individual composition expectation (ICE) plot is superimposed on the PDP. **B** Two cohorts are optimally separated by the SHAP values by applying auto-cohort feature of explanation, utilizing a DecisionTreeRegressor from scikit-learn. By this, separation is given between those scoring less than 1.5 and those $\ge$ 1.5 in the FAQ. Hence, creating two cohorts with 65 and 75 subjects in each. The bar plot displays the mean SHAP values for each group for each feature

The PDP was included to determine the marginal contribution of each of the features to the RF model output. From this, we could estimate the prediction within a range of values associated with an increased likelihood of being defined as cAD. By this, we could estimate the specific effect on the prediction associated with this increased likelihood within the range of values of each feature. Although somewhat artificial, separating the impact of each feature can give insight into the model’s behavior. PDP for the top three ranked features across our importance estimates, the FAQ, RAVLT immediate recall, and hippocampus volume is illustrated in Figs. 6 and 7A. The Y-axis shows changes in the prediction. Positive values represent to what degree a feature increases the likelihood of correct prediction, while a reduction in this likelihood correspond to negative values. As expected, PDP results for the RAVLT immediate recall measure showed that remembering more words lowers the risk of being defined as cAD (Fig. 6A). In most individuals, there seems to be a critical gap between 33 and 40 words, while fewer than 33 words seem to lower its predictive value. Looking at the individual effects from the ICE plots indicates the same tendency as in the PD plots regarding the immediate recall measure. Furthermore, the PDP results of hippocampus volume (eTIV-normalized) indicated that a larger volume tends to decrease the risk of conversion to AD up to 0.005 mm3 (Fig. 6B). The ICE plot displays that most subjects with MCI follow this average prediction pattern. However, some skewed trends seem to appear. Some subjects with a higher probability of being defined as cAD seem to be dependent on a slightly enlarged hippocampus volume before volume appears to provide a protective effect. Thus, these results suggest considerably larger heterogeneity in this measure than in the immediate recall measure.

Finally, the PDP for the FAQ shows that even a slight increase in the FAQ score (1-2) increased the odds of converting from MCI to AD, and from score 5 and onward, there is an above 20% increased chance for such a disease progression (Fig. 7A). The same trend was displayed by the ICE plot. Additionally, Fig. 7B, illustrates two cohorts, with 65 and 75 subjects in each, that are optimally generated by applying auto-cohort SHAP explanation. Without predefined restrictions, consistently with the PDP of FAQ, a separation for defining two groups was automatically given between those scoring less than 1.5 and those scoring $\ge$ 1.5 on the FAQ. Thus, the SHAP values conditioned across these two groups may indicate how the feature may contribute differently to the RFs models’ decision behavior. Accordingly, the subgroup with lower FAQ scores may contribute to better prediction performance across features. The opposite effect seems to matter in the group of subjects with higher FAQ scores. The RAVLT delay recall score in these subjects also seemed more influential for the prediction than in the cohort with lower FAQ scores. Overall, the PDP for FAQ and the SHAP auto-split results underline the importance of subtle changes in the FAQ score for decision behavior in the current RF prediction model.

## Discussion

Results from the RF classification model incorporating k-fold cross-validation, grid-search, and testing on a hold-out test set showed a prediction accuracy of around 70%. Clinical descriptions of correctly classified and misclassified patients included well-known AD traits in patients who were correctly and falsely allocated to the group of patients with MCI who over time were diagnosed with AD. A strong weight was given to everyday functioning, verbal memory, and a volume measure of the hippocampus. This finding was consistently supported across three different permutation-driven estimates of feature importance. To build confidence in the results, we used additional post hoc analyses to investigate which features contributed considerably to the RF model. The post hoc analyses i) showed that even subtle changes in everyday functioning noticed by a close informant put MCI patients at increased risk for an AD diagnosis later in life, ii) showed the robustness of memory function as a clinical predictor and iii) illustrated the heterogeneity of volume measures of the hippocampus.

The FAQ is known as a valid instrument to assess functional activity levels in older adults [15, 27]. However, a questionnaire will always only give an indirect measure of the skills and behavior described by the items. Both over- and under-estimations are therefore expected. Thus, we find it intriguing that FAQ was identified among the top three most important features by the RF model and that three independent permutation estimates confirmed this. An AD diagnosis is defined when cognitive deficits are severe enough to interfere with daily life functioning. Impaired cognitive function is also present in patients with an MCI diagnosis, but this impairment should not interfere with daily life functioning. The present study indicates that this is not always the case. Originally, a FAQ score equal to or above 9 (preferably, but not exclusively, dependent on three or more activities) is used to indicate impaired ADL function [15]. As shown in the confusion matrix (Fig. 2), 10 of 45 MCI patients who later were diagnosed with AD were rated with a score of 9 already at the baseline examination. The mean for this subgroup was 5.7, while the mean FAQ score was much lower (1.8) for both those who were correctly and falsely classified as sMCI. Interestingly, recent studies suggest that the cut-off score for impairment should be 5 or 6 [28]. The results in the present study indicated an even lower threshold value. As displayed in Fig. 7A, the marginal effect of FAQ total score on our RF model indicates that even a slight increase in the FAQ score (1-2) of a patient with MCI elevates the odds of a future AD diagnosis. For example, from a score of 5 points, there is more than 20% increased chance for an MCI patient to be on a path towards an AD diagnosis, and a similar trend can be observed in the superimposed ICE plot. Similarly, the auto-two-cohort split illustrated in Fig. 7B optimally separated the SHAP values between those subjects scoring less than 1.5 (*n*=65) and those ${\ge }$ 1.5 on the FAQ (*n*=75). According to the mean SHAP values, lower FAQ scores may contribute to better prediction performance across features. The opposite was displayed in the cohort of patients with higher FAQ scores, where the performance on the delayed recall test seemed to be more influential. This is in line with previous studies suggesting that while learning is a sensitive diagnostic measure for MCI, poor performance on a delayed recall test puts an MCI patient at higher risk of progressing to dementia [29, 30]. Still, the FAQ was considered to be most important for the prediction also in this subgroup of subjects, all with a FAQ score of ${\ge }$ 1.5. The present results support the inclusion of measures of everyday life activities already at an initial clinical evaluation of patients presenting symptoms indicating MCI [31].

The correctly and misclassified patients’s characteristics also gave some interesting results among the subgroup of sMCI patients who were falsely classified as cAD. A more detailed investigation of sub-items from FAQ (Table S1) showed that this subgroup was impaired on items described as particularly predictive of dementia in previous studies: paying bills (finance), organizing taxes (form), and remembering dates [31]. Thus, further studies should investigate the predictive power of specific clusters of impaired daily life functioning and whether some sub-items are more vulnerable to reporting biases than others. By removing and adding items well adapted to the age and nationality of the patient, one could try to improve the instrument’s validity. Furthermore, in ADNI, patients with clinical depression were excluded from the dataset, motivated by studies showing MCI in patients with depression and that successful treatment of even mild symptoms of depression may reverse an MCI diagnosis [32]. Thus, none of the patients in our sample obtained a GDS score above 5. It is also worth noticing that the highest mean GDS scores are found in the correctly classified sMCI subgroup, and it is only in this subgroup we find subjects with a score of 5 on this depression scale (Fig. 2). Moreover, characteristics of the patients wrongly classified as stable MCI while the true outcome was AD showed a larger hippocampus volume, preserved LVV, and higher cognitive performance level than the correctly classified cAD. On the other hand, those patients falsely classified as converters to AD had a similar pattern of cognitive and structural decrease as the patients correctly predicted as cAD. Overall, these results illustrate the brain-behavior complexity. Although the current study is not appropriate to directly study this relationship, the described profiles of subjects’ classification are in line with the age-related brain maintenance [33, 34] and cognitive reserve hypotheses [35, 36].

Furthermore, the present study supports that impairment of memory function and reduced volumes in related morphometrical brain regions are hallmarks of early stages of AD [37–39]. As expected from previous studies from our group [11, 12], the current results accorded memory function and total hippocampus volume substantial weights in the prediction model. In addition, the present study added information about the relations between these impairments and reports on the FAQ. As illustrated by the SHAP interaction estimates shown in Fig. S1, the performance on FAQ, as well as on measures of memory function, and hippocampus volume, seem to have unique contributions to our classification model. The SHAP summary plot further illustrates that larger deficits in functional activities level, a lower verbal memory function and hippocampus volume improve the prediction of conversion to AD. In contrast, a decrease in this prediction is associated with preserved levels in these measures. These SHAP results are in accordance with the expected decline in ADL, memory-related brain structure, and cognitive function characterizing the dementia of an AD patient and thus give confidence to our RF model decisions. By visual inspections of the dependency plots (Fig. 6), both the general and individual descriptions suggest that a low number of words immediately recalled from a word-list by a patient with MCI increases the odds of converting to an AD diagnosis by about 20% in our model. Most individuals seem to have a critical point between 33 and 40 words. Furthermore, the skewed trend illustrated by the ICE plot for hippocampus volume suggests the inclusion of subjects with somewhat different model behavioral patterns. While the general tendency shown in the PDPs indicated that larger volumes, up to 0.005 mm3, seem to decrease the risk of converting to AD, this may not be the case for all patients. The marginal effect on the prediction showed that a higher probability of converting to AD seems to be dependent on a slightly larger hippocampus volume before it has a protective effect. However, the contribution of hippocampus volume in our RF model should be interpreted with caution. Although the hippocampus is one of the most studied AD-related brain regions, it should not qualify as a stand-alone measure for an early diagnosis of dementia [40].

In that regard, some strengths and limitations should be mentioned. The pre-processing of the MRI data using the longitudinal stream of FreeSurfer [16] is a strength of the present study by increasing the reliability of the extracted hippocampus volume [41]. Combined bilateral brain volumes were also used to provide a reliable measure of the ventricle and hippocampus volumes [42, 43]. However, asymmetrical patterns in hippocampal volumes have been associated with MCI and AD differences [44]. Recent studies have also investigated parcellated subregions of the hippocampus to improve our understanding of this biomarker [45]. Thus, follow-up studies should expand on the present processed data and investigate the predictive values of subcomponents of the hippocampus and other related brain structures such as the entorhinal cortex [46], including both combined and lateralized measures of the brain volumes. In addition, future explorations should also consider the inclusion of a nonparametric Bayesian framework that allow for probability distributions in order to generalize the specific and joint feature prediction contribution [47].

A final remark should be given to the prediction performance and the methodology used in the present study. Here, two groups were defined according to their longitudinally defined diagnosis. We investigated the contribution of information about everyday life functioning in the context of well-defined cognitive and MRI markers in predicting disease outcomes. ADNI holds strict inclusion criteria, aiming to encounter amnestic MCI. MCI is nevertheless regarded as a heterogeneous diagnostic category both in terms of clinical symptoms [48, 49], structural deterioration [50–52], and temporal disease stage progression [53, 54]. A prediction at a modest level by our ML approach should thus be expected. The accuracy achieved in the current study is below what should be considered sufficient to enable direct implementation in clinical practice. Still, we believe that the results should inspire further work toward developing automated prognostic tools within a person-specific multi-factorial clinical framework. A main contribution of the present study was to show that FAQ was selected as one of the most predictive features. In that FAQ takes a relatively short time to administer, it should be easy to implement as part of a comprehensive clinical assessment of patients with mild as severe cognitive impairment. This suggests that the clinical validity of questionnaires like FAQ should be a topic for future research. The present study also contributed by including analytic models needed in the area of precision medicine [24]. Future explorations should go beyond such supervised learning, e.g., by including a functional random forest model [55–57]. Using this and other methods, it should be possible to detect distinct mechanisms in subgroups defined within the diagnosis of MCI. Future models, preferably with higher accuracy levels, should be evaluated also on external validation data [58]. Finally, although the post hoc SHAP exploration supports the general feature importance in our prediction model, model explainability should be interpreted with caution [58, 59]. Approximation of Shapley values, e.g., the active coalition of variables (ACV) proposed by Amoukou and colleagues, should be considered in the context of correlated features [60].

## Conclusion

In conclusion, three different permutation-driven estimates consistently supported the importance of including measures of everyday functioning in a model predicting future conversion to AD. The diverse post hoc ML explorations indicated that even subtle changes in everyday functioning might predict progression to AD at an early stage of the disease. Therefore, this information should be included as part of an initial multi-factorial clinical evaluation of patients with MCI. Information on these changes should also be included in future longitudinal studies of different pathways from normal cognitive aging to the cognitive decline characterizing different stages of AD and other neurodegenerative disorders.

## Supplementary Information

**Additional file 1:** **Supplementary Table 1.** Characteristics of the correctly and miss-classified sMCI and cAD.

**Additional file 2:** **Supplementary Figure 1.** SHAP interaction values. Displays the SHAP interaction values. The main effect of each feature is shown on the diagonal, while interaction effects are shown off-diagonal.

## Data Availability

Data included in this article were obtained from the Alzheimer’s Disease Neuroimaging Initiative (ADNI) database (http://adni.loni.usc.edu). The code to reproduce our results is available via GitHub: https://github.com/marekkoc/Vik_et_al_FAQ-predictor-of-AD_paper-source-code/blob/main/faq-predictor-of-ad.ipynb.

## Abbreviations

ADL: Activities of daily living
AD: Alzheimer’s Disease
ADNI: Alzheimer’s Disease Neuroimaging Initiative
eTIV: Estimated total intracranial volume
FAQ: Functional activity/assessment questioner
ICE: Individual composition expectation
LVV: Lateral ventricle volume
ML: Machine learning
MCI: Mild cognitive impairment
MRI: Magnetic resonance imaging
PDP: Partial dependence plots
RAVLT: Rey Auditory Verbal Learning Test
SHAP: SHapley Additive exPlanations
TMT: Trail Making Test
