# Supplementary material for: Functional activity level reported by an informant is an early predictor of Alzheimer’s disease
Source: BMC Geriatr. 2023 Mar 31;23:205. doi: 10.1186/s12877-023-03849-7 (PMC10067216; doi:10.1186/s12877-023-03849-7)
Supplement: Supplementary file 1 — Additional file 1: Supplementary Table 1. Characteristics of the correctly and miss-classified sMCI and cAD. [file 12877_2023_3849_MOESM1_ESM.pdf]

Table 1. Characteristics of the correctly and miss-classified sMCI and cAD.

	False Negative	False Positive	True Negative	True Positive
Demographics				
N	20	17	58	45
Sex (F:M)	8:12	7:10	25:33	17:28
Age [years]: mean (SD)	74 (8.3)	76 (5.5)	72 (7.5)	74 (7.0)
Education [years]: mean (SD)	16.1 (2.5)	16.8 (3.5)	16.0 (2.9)	15.4 (2.8)
Participation length [years]: mean (SD)	6.1 (2.8)	3.4 (2.7)	4.8 (2.7)	5.3 (2.9)
Cognitive function				
RAVLT immediate recall: mean number (SD)	37.2 (6.7)	29.1 (6.2)	39.1(8.7)	27.8(4.8)
RAVLT delayed: mean number (SD)	4.3 (3.2)	1.2 (1.3)	5.7 (3.5)	1.5 (1.9)
RAVLT recognition: mean number (SD)	11.6 (2.6)	9.0 (3.6)	12.1 (2.6)	9.0 (3.7)
TMTA: mean seconds (SD)	41 (29)	43 (8)	37 (12)	45 (25)
TMTB: mean seconds (SD)	115 (82)	131 (56)	93 (36)	140 (80)
CFT animals: mean number (SD)	16.9 (4.7)	16.2 (5.6)	18.7 (4.7)	15.2 (4.1)
MRI measures				
LVV (eTIV normalized): mean (SD)	0.025 (0.010)	0.030(0.011)	0.023 (0.011)	0.030 (0.015)
Hippocampus (eTIV normalized): mean (SD)	0.0045 (0.00)	0.0037(0.00)	0.0046 (0.00)	0.0038 (0.00)
Functional levels				
GDS: mean (SD)	1.5 (1.2)	1.6 (0.9)	1.9 (1.4)	1.4 (1.2)
FAQ Total: mean (SD)	1.8 (2.3)	4.4 (4.8)	1.8 (3.7)	5.7 (4.8)
FAQ Bills: mean (SD)	0.2 (0.4)	1.0 (1.1)	0.3 (0.7)	0.6 (0.8)
FAQ Taxes: mean (SD)	0.2 (0.5)	0.8 (0.9)	0.2 (0.6)	1.1 (1.0)
FAQ Shopping: mean (SD)	0.1 (0.3)	0.3 (0.7)	0.1 (0.4)	0.4 (0.7)
FAQ Games: mean (SD)	0.3 (0.7)	0.4 (0.8)	0.1 (0.4)	0.4 (0.6)
FAQ Beverage/Stove: mean (SD)	0.0 (0.0)	0.1 (0.5)	0.0 (0.4)	0.1 (0.3)
FAQ Meal Prep: mean (SD)	0.3 (0.6)	0.1 (0.3)	0.1 (0.5)	0.4 (0.6)
FAQ Events: mean (SD)	0.2 (0.5)	0.2 (0.6)	0.1 (0.4)	0.5 (0.9)
FAQ Pay Attention (TV): mean (SD)	0.2 (0.4)	0.4 (0.6)	0.2 (0.4)	0.4 (0.6)
FAQ Remember Dates: mean (SD)	0.4 (0.7)	0.8 (0.8)	0.3 (0.7)	1.1 (0.8)
FAQ Travel: mean (SD)	0.2 (0.5)	0.4 (0.6)	0.2 (0.7)	0.7 (0.8)

RAVLT: Rey Auditory Verbal Learning Test, TMT: Trail Making Test part A and B, CFT: Category Fluency Test; LVV: lateral ventricle volumes, GDS: Geriatric Depression Scale, FAQ: Functional Activity Questioner, False negative (FN): predicted sMCI while observed (true) outcome cAD, False Positive (FP): predicted cAD when observed (true) outcome is sMCI, True Negative (TN): predicted and observed (true) outcome is sMCI, True Positive (TP): predicted and observed (true) outcome is cAD.
