# Supplementary figures and images for: Functional activity level reported by an informant is an early predictor of Alzheimer’s disease
Source: BMC Geriatr. 2023 Mar 31;23:205. doi: 10.1186/s12877-023-03849-7 (PMC10067216; doi:10.1186/s12877-023-03849-7)

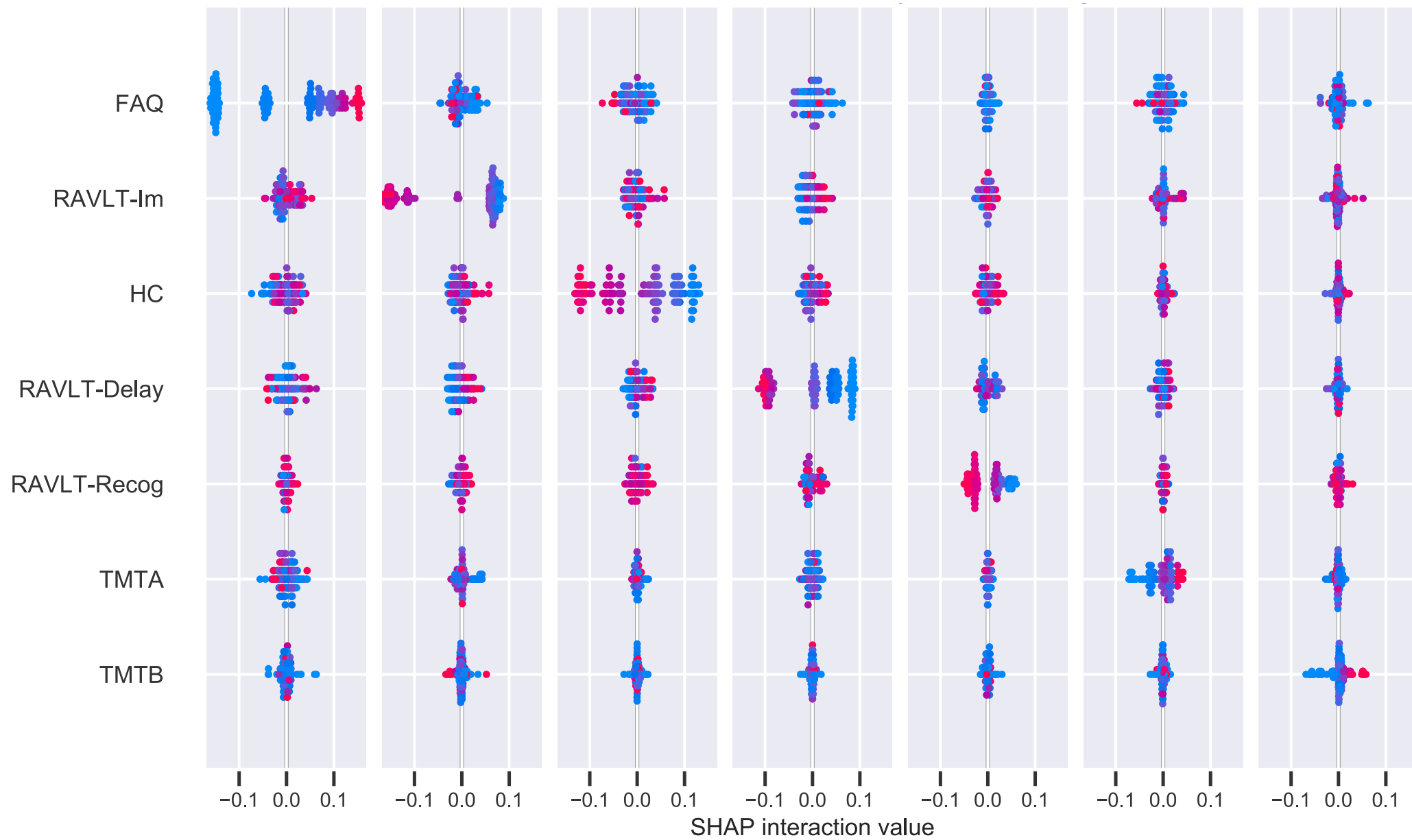

Supplement: Supplementary file 2 — Additional file 2: Supplementary Figure 1. SHAP interaction values. Displays the SHAP interaction values. The main effect of each feature is shown on the diagonal, while interaction effects are shown off-diagonal. [file 12877_2023_3849_MOESM2_ESM.pdf]
